# Munc13-Like skMLCK Variants Cannot Mimic the Unique Calmodulin Binding Mode of Munc13 as Evidenced by Chemical Cross-Linking and Mass Spectrometry

**DOI:** 10.1371/journal.pone.0075119

**Published:** 2013-10-10

**Authors:** Sabine Herbst, Daniel Maucher, Marian Schneider, Christian H. Ihling, Olaf Jahn, Andrea Sinz

**Affiliations:** 1 Department of Pharmaceutical Chemistry & Bioanalytics, Institute of Pharmacy, Martin Luther University Halle-Wittenberg, Halle (Saale), Germany; 2 Research Group Artificial Binding Proteins, Institute of Biochemistry and Biotechnology, Martin Luther University Halle-Wittenberg, Halle (Saale), Germany; 3 Proteomics Group, Max-Planck-Institute of Experimental Medicine, Göttingen, Germany; California Institute of Technology, United States of America

## Abstract

Among the neuronal binding partners of calmodulin (CaM) are Munc13 proteins as essential presynaptic regulators that play a key role in synaptic vesicle priming and are crucial for presynaptic short-term plasticity. Recent NMR structural investigations of a CaM/Munc13-1 peptide complex have revealed an extended structure, which contrasts the compact structures of most classical CaM/target complexes. This unusual binding mode is thought to be related to the presence of an additional hydrophobic anchor residue at position 26 of the CaM binding motif of Munc13-1, resulting in a novel 1-5-8-26 motif. Here, we addressed the question whether the 1-5-8-26 CaM binding motif is a Munc13-related feature or whether it can be induced in other CaM targets by altering the motif's core residues. For this purpose, we chose skeletal muscle myosin light chain kinase (skMLCK) with a classical 1-5-8-14 CaM binding motif and constructed three skMLCK peptide variants mimicking Munc13-1, in which the hydrophobic anchor amino acid at position 14 was moved to position 26. Chemical cross-linking between CaM and skMLCK peptide variants combined with high-resolution mass spectrometry yielded insights into the peptides' binding modes. This structural comparison together with complementary binding data from surface plasmon resonance experiments revealed that skMLCK variants with an artificial 1-5-8-26 motif cannot mimic CaM binding of Munc13-1. Apparently, additional features apart from the spacing of the hydrophobic anchor residues are required to define the functional 1-5-8-26 motif of Munc13-1. We conclude that Munc13 proteins display a unique CaM binding behavior to fulfill their role as efficient presynaptic calcium sensors over broad range of Ca^2+^ concentrations.

## Introduction

Calmodulin (CaM) is a small acidic protein and is one of the most prominent Ca^2+^ sensors. It is ubiquitously found and extraordinarily conserved from yeast to human [1]. CaM binds to a large number of different intracellular proteins in a Ca^2+^ dependent or independent manner, including kinases, phosphatases, cytoskeletal proteins, and transcription factors. One important example of neuronal CaM targets is present in Munc13 proteins, which constitute essential presynaptic regulators. Munc13 proteins play a key role in synaptic vesicle priming and are involved in presynaptic short-term plasticity (STP) [2], [3], [4]. The binding of Munc13 to CaM was found to link residual Ca^2+^ signaling with the synaptic exocytotic machinery [5]. All Munc13 isoforms share a highly conserved *C*-terminal region, but possess divergent *N*-termini with the CaM binding sites (see [6] for overview). Munc13 peptides, comprising the CaM binding regions, form 1∶1 complexes with CaM already at submicromolar Ca^2+^ concentrations [7]. Recently, we applied complementary cross-linking strategies to gain first insights into the 3D-structures of Munc13/CaM complexes and showed that 21-amino acid Munc13 peptides bind the *C*-terminal CaM domain through a classical antiparallel 1-5-8 CaM binding motif with the numbers indicating the hydrophobic anchor residues of the motif [8], [9].

Yet, a second interaction site was identified in the homologous Munc13 isoforms Munc13-1 and ubMunc13-2 when CaM binding of *C*-terminally elongated 34-amino acid Munc13-1 and ubMunc13-2 peptides was studied by the complementary techniques of photo-affinity labeling (PAL) and NMR spectroscopy. Thereby, it was found that a tryptophan at position 26 of the CaM binding motif anchors the *C*-termini of Munc13 peptides to the *N*-terminal CaM domain, creating a novel, so-called “1-5-8-26 motif” with hydrophobic anchor amino acids at positions 1, 5, 8, and 26 [10]. This motif is conserved in Munc13-1 and ubMunc13-2 homologues from almost all species analyzed so far. The NMR structure of the CaM/Munc13-1 peptide complex (PDB entry 2KDU) revealed a unique binding behavior of this longer Munc13-1 peptide to CaM, with the peptide exhibiting an α-helical part, such as in classical CaM targets, in addition to a hairpin structure with a second CaM binding site. In this complex, CaM shows an extended structure, which greatly differs from the structure that CaM exhibits in its complexes with classical CaM targets in the presence of calcium.

In the present study, we investigated whether such a 1-5-8-26 CaM binding motif is unique for Munc13-1 and ubMunc13-2 or whether it can be principally generated from other 1-5-8-based motifs by *C*-terminal elongation and specific amino acid exchanges. For this purpose, we chose the well characterized CaM target skeletal muscle myosin light chain kinase (skMLCK) as a prototype of the 1-5-8-14 family of CaM binding motifs and modified it to mimic the sequence of Munc13.

**Figure 1:**
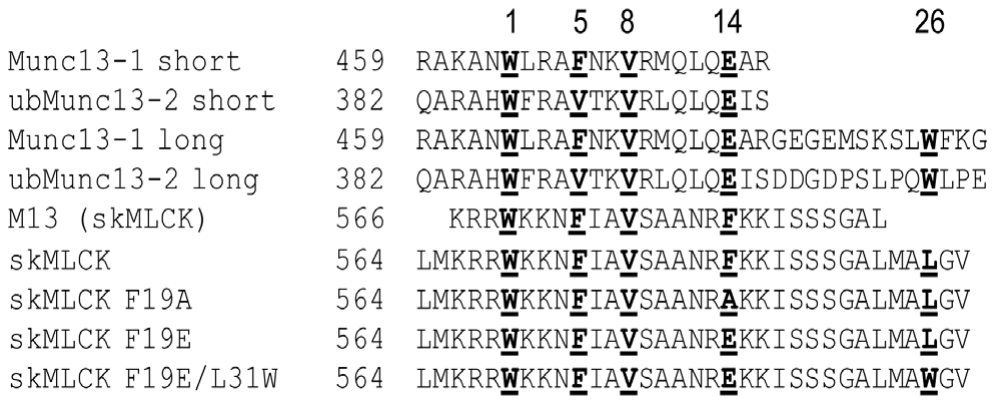
Amino acid sequences of peptides representing the CaM binding motifs of Munc13-1 and skMLCK. Numbers of *N*-terminal amino acids refer to full length proteins. The peptides used in this study are skMLCK, skMLCK F19A, skMLCK F19E and skMLCK F19E/L31W. Hydrophobic anchor amino acids are shown in ***bold and underlined,*** the numbers above the amino acid sequences indicate their respective positions in the CaM binding motif.

The interaction between CaM and skMLCK is crucial for muscle contraction. Upon Ca^2+^ binding, CaM activates skMLCK by interacting with the autoinhibitory sequence of the protein and, thereby, inducing a domain movement. This allows skMLCK to bind to its substrate myosin and to exert its function as kinase [11], [12]. The NMR structure of the CaM/skMLCK peptide complex [13] shows that CaM collapses around the 26-amino acid skMLCK peptide, called M13, comprising the CaM binding region (Figure 1). The binding behavior of the skMLCK peptide M13 to CaM in an antiparallel manner according to a 1-5-8-14 motif is comparable to that of short (21-amino acid) Munc13 peptides [8]. Therefore, the question arises, how the complex between CaM and the *C*-terminally elongated skMLCK peptides will be configured – collapsed or extended – in case they are modified to mimic Munc13's 1-5-8-26 motif.

In order to address this question we synthesized four skMLCK peptides containing a *C*-terminally elongated CaM binding region with single amino acid exchanges at positions 14 and 26 of the CaM binding motif (Figure 1). Phe-19 of the skMLCK peptide corresponds to position 14 of the CaM binding motif and replacement of Phe-19 with an acidic glutamate residue mimics the respective amino acid (Glu) in the Munc13-1 peptide. The additional exchange of Leu-31 (corresponding to position 26 in the CaM binding motif) to tryptophan results in the 1-5-8-26 motif found in Munc13-1 and ubMunc13-2. For gaining detailed information about the binding modes of the four skMLCK peptide variants to CaM (Figure 1) we used chemical cross-linking in combination with high-resolution mass spectrometry – a technique that has matured into an alternative approach to derive structural information of protein complexes [14], [15], [16]. Cross-linking experiments were conducted with two cross-linkers yielding complementary information: The homobifunctional amine-reactive, isotope-labeled cross-linker BS^2^G (*bis*(sulfosuccinimidyl)glutarate) with two *N*-hydroxysuccinimide (NHS) ester sites and the heterobifunctional amine/photo-reactive cross-linker SBC (*N-*succinimidyl-*p*-benzoyldihydrocinnamate) (Scheme S1 in File S1). Additionally, we performed surface plasmon resonance (SPR) measurements to investigate the effect of amino acid exchanges in the CaM binding motif on the peptides' binding affinities.

## Experimental Procedures

### Proteins and Reagents

CaM (bovine brain) was obtained from Calbiochem (Bad Soden, Germany) and used without further purification. CaM was confirmed by MALDI-TOF-MS to be *N*-terminally acetylated and trimethylated at Lys-115; skMLCK peptides were synthesized by standard fluorenylmethoxycarbonyl (Fmoc) chemistry as previously described [17]. The isotope-labeled cross-linking reagent *bis*(sulfosuccinimidyl)glutarate (BS^2^G-*D_0_* (non-deuterated) and BS^2^G-*D_4_* (four-times deuterated)) was purchased from Thermo Fisher Scientific (Darmstadt, Germany); *N*-succinimidyl-*p*-benzoyldihydrocinnamate (SBC) was synthesized *in-house* [18] (Scheme S1 in File S1). Trypsin (porcine, mass spectrometry grade or sequencing grade modified) and the trypsin enhancer ProteaseMax™ Surfactant were purchased from Promega (Mannheim, Germany). Nano-HPLC solvents were spectroscopic grade (Uvasol, VWR, Darmstadt, Germany). Water was purified with a Pacific-UP/UPW water purification system (Thermo Fisher Scientific-TKA, Niederelbert, Germany). All other chemicals were purchased from Sigma (Taufkirchen, Germany).

### Chemical Cross-linking

For all cross-linking reactions, CaM was diluted to 10 µM in 20 mM HEPES buffer (pH 7.2) containing a Ca^2+^/chelator (EGTA) system, with free Ca^2+^ concentrations adjusted between 0 nM to 50 mM as described previously [18]. Initially, different calcium concentrations were used in a calcium titration experiment with BS^2^G-*D_0_* (Scheme S1 in File S1). The diluted CaM solution (10 µM) was mixed with 10 µM of the respective skMLCK peptide (in 20 mM HEPES, pH 7.2) and incubated for 15 min. To start the cross-linking reaction, BS^2^G (in DMSO) was added in a 50-fold molar excess to the protein solution. After an incubation time of 60 min, the reaction was quenched by adding NH_4_HCO_3_ to a final concentration of 20 mM. For the cross-linking reactions with BS^2^G presented herein, Ca^2+^ concentrations of 30 nM and 1 mM were used. The workflow was the same as for the Ca^2+^ titration experiment described above, except that BS^2^G was used in a *D_0_*/*D_4_* (1∶1) mixture to facilitate MS identification of cross-linked products by their characteristic isotope patterns. The cross-linking reactions with SBC were conducted in a two-step fashion [18]. First, the amine-reactive NHS ester site of SBC was allowed to react with CaM for 30 min after adding a 50-fold molar excess of a freshly prepared solution of SBC in DMSO to the 10 µM CaM solution. Quenching of non-reacted NHS ester sites of SBC was carried out with NH_4_HCO_3_ (20 mM final concentration). Afterwards, non-reacted SBC was removed by microfiltration with YM-10 centrifugation units or Amicon Ultra centrifugal units (10 K) (Millipore, Schwalbach, Germany). For the photo-cross-linking reaction, the respective skMLCK peptide (10 µM) was mixed with SBC-labeled CaM before irradiating the mixture with UV-A light (maximum at 365 nm) in a home-built device [19]. 200 µl-aliquots were taken at irradiation energies of 4 and 8 J/cm^2^ and concentrated by microfiltration as described above. The eluates were stored at −20°C before MS analysis was performed.

### MALDI-TOF-MS of CaM/skMLCK Peptide Complexes

After desalting the solutions containing the cross-linked CaM/peptide complexes (ZipTip C_4_, Millipore, Schwalbach, Germany), 1.2 µl of the respective solution was mixed with 1.2 µl of 2,5-dihydroxybenzoic acid (DHB, Sigma) as matrix (20 mg/ml) in 50% (v/v) acetonitrile (ACN) and 0.1% (v/v) trifluoroacetic acid (TFA) and prepared on a steel target. MALDI-TOF-MS measurements were conducted on an Ultraflex III MALDI-TOF/TOF mass spectrometer (Bruker Daltonik, Bremen, Germany) in linear and positive ionization mode. Spectra in the *m/z* range 7,000–33,000 were acquired using FlexControl version 3.3 and processed with FlexAnalysis version 3.3 (Bruker Daltonik).

### Gel Electrophoresis and In-Gel Digestion

Cross-linked CaM/skMLCK peptide complexes were separated from non-reacted CaM using one-dimensional sodium dodecyl sulfate polyacrylamide gel electrophoresis (SDS-PAGE) (15% resolving gel) [20]. After staining with Coomassie Brilliant Blue R250, gel bands of interest were excised and washed as published previously [21]. *In-gel* digestion with trypsin (240 ng per gel band) and the trypsin enhancer ProteaseMax™ Surfactant was performed according to the manufacturer's instructions for 2 hours at 37°C.

### Nano-HPLC/nano-ESI-LTQ-Orbitrap-MS/MS

Tryptic peptide mixtures were fractionated on an Ultimate nano-HPLC system equipped with Famos autosampler and Switchos II (LC-Packings/Dionex, Idstein, Germany) using a trapping column (Acclaim PepMap, C18, 100 µm×20 mm, 5 µm, 100 Å, Dionex) for desalting (15 min, 0.1% (v/v) TFA) followed by a separation column (Acclaim PepMap, C18, 75 µm×200 mm, 3 µm, 100 Å, Dionex) with a 90-min gradient of 0–50% B (A: 5% (v/v) ACN containing 0.1% (v/v) formic acid (FA); B: 80% (v/v) ACN containing 0.08% (v/v) FA). The nano-HPLC system was directly coupled to the nano-ESI source (Proxeon, Odense, Denmark) of an LTQ-OrbitrapXL mass spectrometer (Thermo Fisher Scientific, Bremen, Germany) that was operated in positive ionization mode. Data dependent MS/MS (collision-induced dissociation, CID) experiments (isolation window 2.5 Th) were conducted in the linear ion trap (LTQ) of the five most abundant signals of the full MS scan in the orbitrap (R = 60,000). Detection of less abundant peaks was enabled using dynamic exclusion (exclusion duration 120 s, after three repeats of fragmentation of one precursor). Data acquisition was controlled via XCalibur 2.0.7 (Thermo Fisher Scientific, Bremen, Germany) in combination with DCMS link 2.0 (Dionex, Idstein, Germany).

### Analysis of Cross-linked Products

Cross-linked products were identified by analyzing MS and MS/MS data with the *in-house* software StavroX 2.0.6. [22] and XCalibur 2.0.7 (Thermo Fisher Scientific, Bremen, Germany). Generation of protein structures and docking of apo-CaM (PDB entry 2L53, aa 1–77; [23]) with holo-CaM (PDB entry 2BBM, aa 78–148; [13]) was performed with open-source Pymol 0-99rc6 (DeLano Scientific, San Francisco).

### CD Spectroscopy

Peptides were dissolved in phosphate buffer (100 mM K_2_HPO_4_/KH_2_PO_4_, pH 7.5) at a concentration range of 70–130 µM and final concentrations were determined spectrophotometrically at 280 nm. Far-UV CD measurements in the presence of increasing 2,2,2-trifluoroethanol (TFE) concentrations were performed at 4°C in a 0.5 mm fused silica cuvette on a Jasco J-810 spectropolarimeter. Up to 20 single far-UV CD spectra were accumulated from 200 to 260 nm with a scan rate of 20 nm/min, 4 s-response, and a band width of 1 nm.

### Surface Plasmon Resonance (SPR) Spectroscopy

The affinities of the different skMLCK variants for CaM were examined by SPR measurements using a Biacore T100 SPR instrument (GE Healthcare, Freiburg, Germany); skMLCK peptides were *N*-terminally biotinylated using EZ-Link NHS-LC-Biotin (Thermo Fisher Scientific, Darmstadt, Germany) with peptide: biotinylation reagent ratios ranging between 1∶10 and 10∶1 (*w/w*). The biotinylation reaction was conducted in PBS buffer (pH 6.5) for 30 min on ice, excess of NHS-LC-Biotin was quenched with 10–20 mM NH_4_HCO_3_ (final concentration), and removed by microfiltration using Amicon Ultra centrifugal units (10 kDa cutoff, Millipore, Schwalbach, Germany). MALDI-TOF-MS was used to check for *N*-terminal biotinylation of all skMLCK peptide variants. The peptides were immobilized on a Series S Sensor Chip SA (GE Healthcare, Freiburg, Germany), which was previously conditioned by three consecutive 1-min injections of a solution containing 1 M NaCl and 50 mM NaOH. Peptide solutions were injected at a flow rate of 5 µl/min until an immobilization level of 350 response units (RU) was reached. For the skMLCK F19E peptide, the maximum immobilization level was 250 RU. CaM was diluted in running buffer (20 mM HEPES buffer (pH 7.4) containing 150 mM NaCl, 1 mM CaCl_2_, and 0.05% (*v/v*) Tween20) to concentrations between 100 pM and 750 nM and injected over the chip surface at a flow rate of 12 µl/min for 1750 s. Running buffer was used as blank. Regeneration of the chip was performed with 20 mM HEPES buffer (pH 7.4) containing 10 mM EGTA and 0.05% (*v/v*) Tween20 for 2 min at a flow rate of 12 µl/min. The temperature was set to 25°C. Response data were double referenced and averaged over 10 s at the end of the association injection to calculate steady-state responses. K_D_ values were determined by fitting equation 1 to steady-state data using SigmaPlot 11.0 (Systat Software, Inc.)
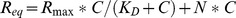
(1)with *R_eq_*: steady-state response, *K_D_*: equilibrium dissociation constant, *C*: CaM concentration, *R_max_*: saturation binding response of high-affinity binding phase, and *N:* slope of observed linear binding phase.

## Results

### Design of skMLCK Peptide Variants

For comparing the binding behavior between Munc13 and CaM to that of the classical CaM target skMLCK, we chose a 33-amino acid sequence stretch representing the CaM binding region of skMLCK. Compared to the so called M13 peptide, comprising solely the CaM binding region of skMLCK [13], the skMLCK peptides used herein are *C*-terminally elongated and contain specific amino acid exchanges at position(s) 14 or/and 26 of the CaM binding motif (corresponding to positions 19 and 31 in the amino acid sequence; Figure 1). Classical CaM binding motifs are 1-5-8-14 motifs with hydrophobic anchor amino acids at positions 1, 5, 8, and 14. However, Munc13-1 and ubMunc13-2 exhibit a 1-5-8-26 motif, which results in an exceptional CaM binding behavior [10]. Therefore, we aimed to address the question whether removal of the anchor amino acid at position 14 together with a second interaction site at position 26 of the CaM binding motif – as in Munc13 peptides – can induce a structure in a classical CaM/target complex comparable to that of the CaM/Munc13-1 complex. The skMLCK peptide was converted in this respect that the hydrophobic anchor phenylalanine at position 14 of the CaM binding motif was exchanged to alanine and glutamate, respectively. Additionally, the leucine residue at position 26 of the motif was exchanged to a comparably hydrophobic, but more bulky tryptophan residue. A glutamate at position 14 and a tryptophan at position 26 strongly resemble the Munc13-1 CaM binding motif (Figure 1).

### Secondary Structures of skMLCK Peptide Variants

Usually, CaM target motifs possess a high propensity to form α-helices - as do the Munc13 peptides [24]. Therefore, we performed CD measurements to compare the secondary structures of skMLCK peptide variants among themselves as well as with those of the Munc13 peptides [9]. As expected, an α-helical structure is induced in the *C*-terminally elongated skMLCK peptide in the presence of increasing TFE concentrations, reaching a maximum α-helical content at 40% TFE (Figure S1A in File S1). CD studies conducted with skMLCK F19A and skMLCK F19E revealed similar results (Figure S1B, C in File S1), indicating that an amino acid exchange at position 14 of the skMLCK CaM binding motif does not result in major changes in secondary structure. On the other hand, CD measurements confirmed a dramatically reduced propensity of skMLCK F19E/L31W to form an α-helical structure (Figure S1D in File S1), as secondary structure prediction (PSIPRED) had indicated. These findings demonstrate that the exchange of Leu-31 in the skMLCK peptide to a bulky hydrophobic tryptophan causes remarkable structural changes in the peptide, raising the question whether this has an influence on the structure of the resulting CaM/peptide complex.

### Ca^2+^ Dependence of CaM/skMLCK Interaction

**Figure 2:**
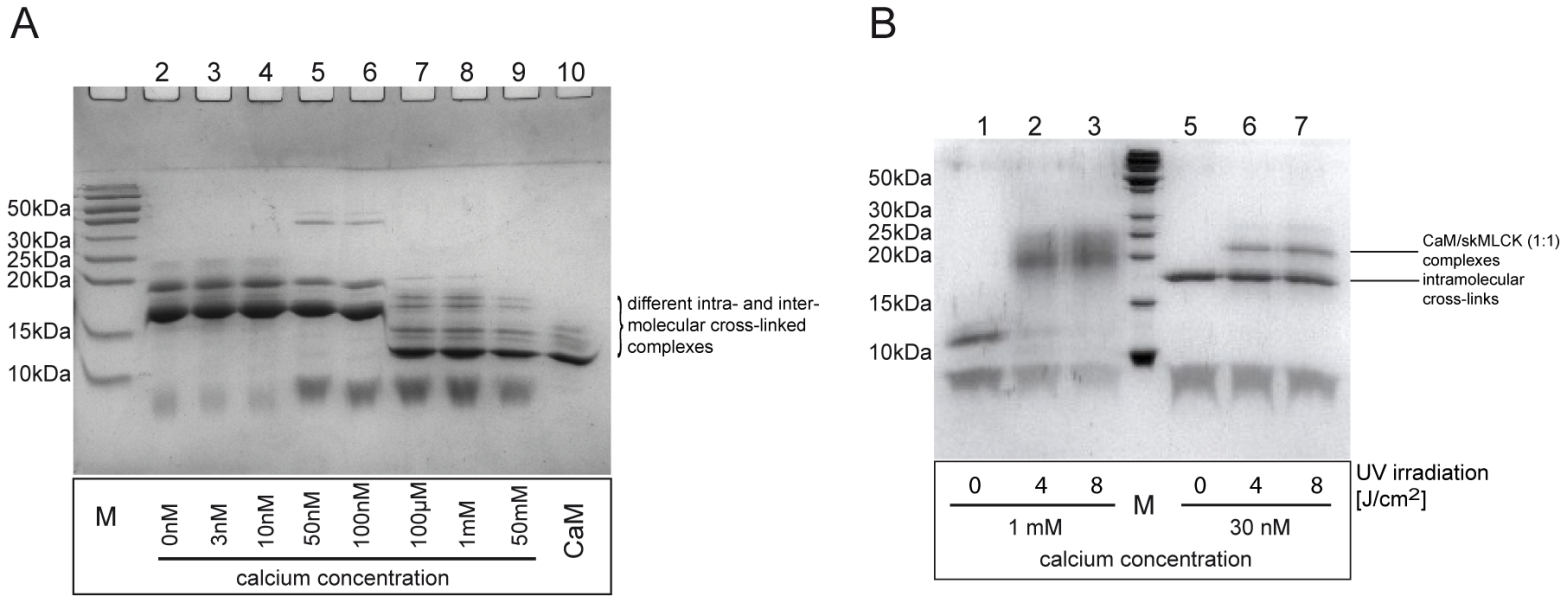
SDS-PAGE of cross-linking reaction mixtures between CaM and skMLCK peptides. (A) Cross-linking reactions between CaM and skMLCK peptide (50-fold molar excess of BS^2^G, Ca^2+^ concentrations: 0 to 50 mM). As a control, CaM was diluted in water without adding buffer, EGTA, or Ca^2+^; please note the different Ca^2+^-loaded states of CaM (lane 10). (B) Cross-linking reactions between CaM and the skMLCK F19E/L31W peptide, conducted at 50-fold molar excess of SBC. Lanes 1–3: 1 mM Ca^2+^; lanes 5–7: 30 nM Ca^2+^. M: protein marker.

Before conducting the cross-linking experiments for 3D-structural analysis of CaM/peptide complexes, we performed Ca^2+^ titration experiments to investigate the influence of Ca^2+^ on CaM/skMLCK peptide complex formation. Figure 2A shows an SDS gel of cross-linking reactions with Ca^2+^ concentrations ranging from 0 to 50 mM. It is readily visible that 1∶1 complexes between CaM and the skMLCK peptide are created with the cross-linker BS^2^G both at low and higher Ca^2+^ concentrations; yet, the CaM/skMLCK peptide complexes created at resting, i.e., nanomolar, Ca^2+^ concentrations seem to be different from those created at higher Ca^2+^ concentrations [25]. A dramatic change in the electrophoretic mobility of CaM is detected at Ca^2+^ concentrations between 100 nM and 100 µM due to a conformational change in CaM upon calcium binding (K_D_ (*C*-terminal lobe of CaM): 2 · 10^−7^ M; K_D_ (*N*-terminal lobe of CaM): 2 · 10^−6^ M [26]). Moreover, at higher Ca^2+^ concentrations (>100 µM) there are several bands visible corresponding to different cross-linked species (both intramolecular cross-links in CaM as well as intermolecular cross-links between CaM and the peptide) exhibiting slightly different electrophoretic mobilities. Subsequent cross-linking experiments were conducted at two different Ca^2+^ concentrations, 1 mM and 30 nM (Figure 2B and Figure S2 in File S1), in order to investigate the binding behavior of skMLCK peptides to CaM both in the low and the high calcium range.

### Cross-linking of CaM/skMLCK Complexes

The cross-linking reactions were conducted with the homobifunctional amine-reactive cross-linker BS^2^G, which reacts mainly with lysine residues, but also with serines, tyrosines, or threonines [27], [28]. Additionally, we used the heterobifunctional amine/photo-reactive cross-linker SBC [18]. The photo-reaction is less specific compared to that of NHS esters and yields a number of potential cross-linking sites. Although the benzophenone moiety might preferably react with methionines after irradiation with long-wavelength UV light (∼365 nm) [18], [29], alternative residues, such as Ala, Phe, Ile, Leu, Lys, and Arg, are susceptible for the cross-linking reaction with SBC. This presents a distinct advantage for investigating protein interaction sites as a less specific cross-linking reaction will result in a greater variety of cross-linked products.

**Figure 3:**
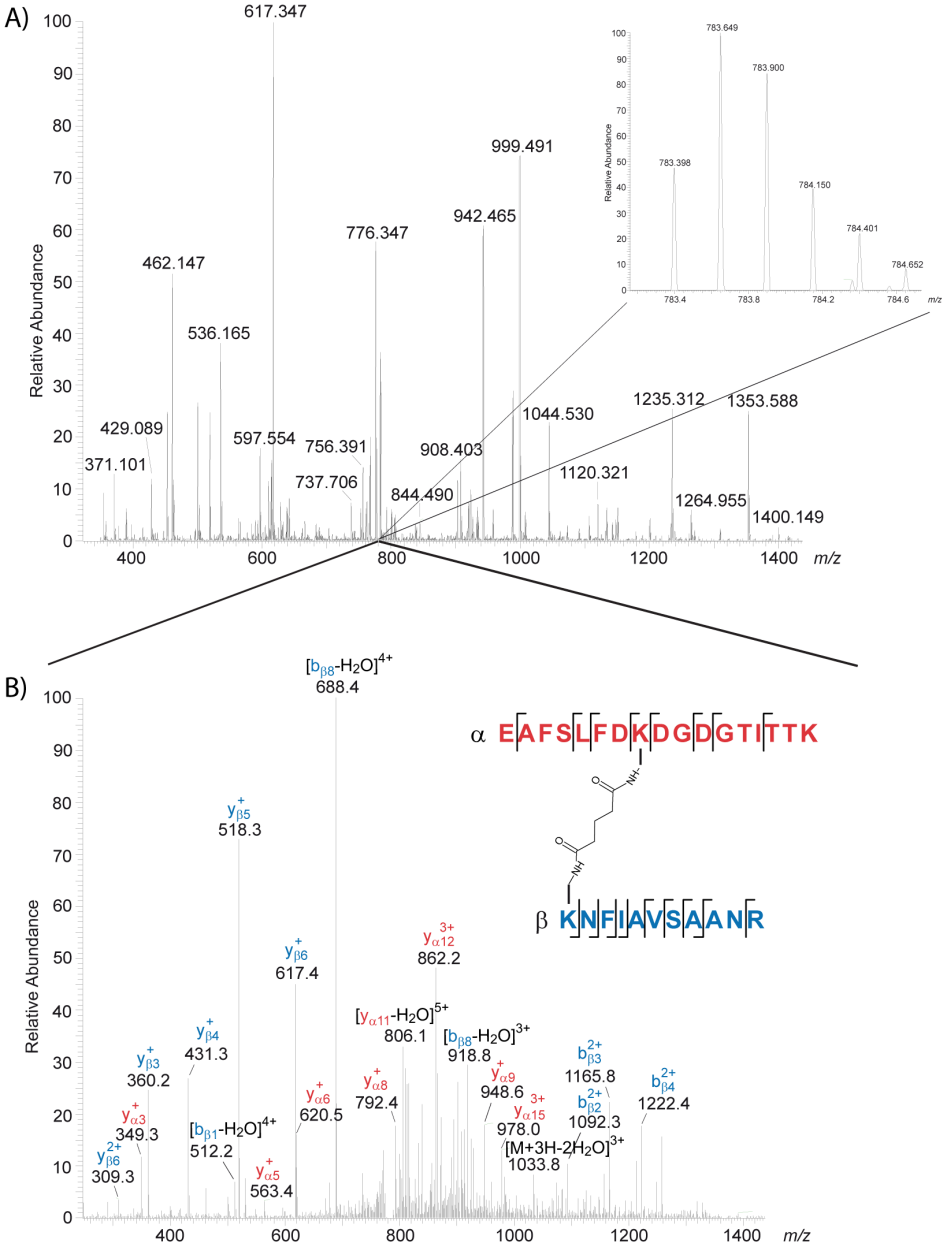
Nano-HPLC/nano-ESI-LTQ-Orbitrap-MS/MS analysis of a cross-linked peptide mixture between CaM and skMLCK peptide. The reaction was conducted at 1^2+^ with a 50-fold molar excess of BS^2^G for 60 min. (A) Mass spectrum (MS) obtained at an LC retention time of 70.42 min. The 4+ charged signal of a cross-linked product at *m/z* 783.398 is shown enlarged. (B) Fragment ion mass spectrum (CID-MS/MS). The cross-linked product comprises amino acids 14–30 of CaM (α-peptide in red) and amino acids 8–18 of skMLCK (β-peptide in blue), in which Lys-21 of CaM is connected to Lys-8 of skMLCK.

**Figure 4:**
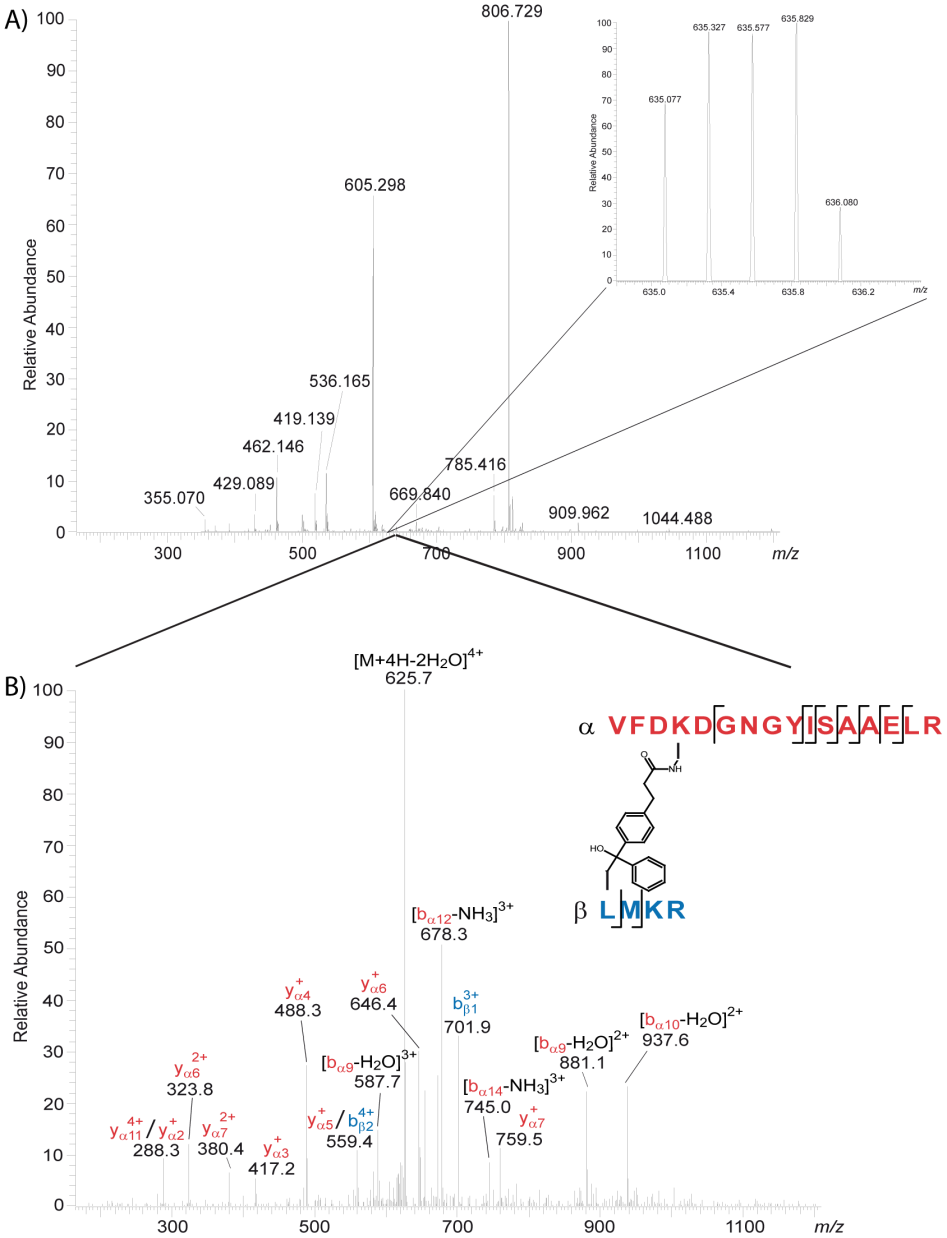
Nano-HPLC/nano-ESI-LTQ-Orbitrap-MS/MS analysis of a cross-linked peptide mixture between CaM and skMLCK F19E/L31W peptide. The reaction was conducted at 1^2+^ with a 50-fold molar excess of SBC (30 min, irradiation 8 J/cm^2^,). (A) Mass spectrum (MS) obtained at an LC retention time of 59.94 min. The 4+ charged signal of a cross-linked product at *m/z* 635.077 is shown enlarged. (B) Fragment ion mass spectrum (CID-MS/MS). The cross-linked product comprises amino acids 91–106 of CaM (α-peptide in red) and amino acids 1–4 of skMLCK F19E/L31W (β-peptide in blue), in which Lys-94 of CaM is connected to Leu-1 of skMLCK F19E/L31W.

Intact BS^2^G-cross-linked CaM/skMLCK peptide complexes were analyzed by MALDI-TOF mass spectrometry (Figure S3 in File S1), showing a reasonable amount of cross-linked CaM/skMLCK peptide (1∶1) complex at 1 mM Ca^2+^. This was confirmed by analyzing BS^2^G- and SBC-cross-linked complexes by gel electrophoresis (SDS-PAGE), with reactions conducted at Ca^2+^ concentrations of 1 mM and 30 nM (Figure 2B). CaM modified with partially hydrolyzed cross-linker (in the case of BS^2^G) as well as intramolecularly cross-linked CaM possess molecular weights around 17 kDa. These various cross-linker-modified species exhibit different electrophoretic mobilities in SDS-PAGE analysis, resulting in several bands in the presence of high Ca^2+^ concentrations (Figure 2A). At low Ca^2+^ concentrations (0–100 nM), two distinct bands are visible for intramolecularly cross-linked CaM and CaM/skMLCK peptide (1∶1) complexes (Figures 2A, B; Figure S2 in File S1). The bands of cross-linked CaM/skMLCK peptide (1∶1) complexes and of cross-linker modified and/or intramolecularly cross-linked CaM were excised from the gel, subjected to *in-gel* digestion, and analyzed by nano-HPLC/nano-ESI-LTQ-Orbitrap-MS/MS (Figures 3 and 4). The number of identified intra- and intermolecular cross-linked products varied between different cross-linking experiments (Table 1 and Tables S1, S2, S3, and S4 in File S1).

**Table 1:** Summary of cross-linked products (intra- and intermolecular) for CaM and skMLCK F19E/L31W peptide.

*m/z* measured	[M+H]^+^_theor._	[Ca^2+^]	CaM	skMLCK E19/W31	Sample
527.507	2107.009	1 mM	K77	L1, M2	SBC4
596.051	2381.185	1 mM	K94	L1, M2	SBC4, SBC8
603.552	2411.188	1 mM	K21	R4, R5	SBC8
608.551	2431.185 (1 Met oxid.)	1 mM	K13	K3	BS^2^G
618.557	2471.205	30 nM, 1 mM	K21	L1, M2	SBC4, SBC8
621.296	2482.161	30 nM	K77	L1, M2	SBC8
635.077	2537.286	1 mM	K94	L1	SBC8
870.757	2610.258	30 nM/1 mM	K75	L1, M2	SBC4
657.582	2627.306	1 mM	K21	L1, M2	SBC4, SBC8
1044.802	3132.389 (1Met oxid.)	30 nM/1 mM	K148	L1, M2	SBC4, SBC8
640.132	3196.633	30 nM	K94	L1, M2	SBC4
828.168	3309.654	30 nM	K94	A12, V13	SBC4
850.674	3399.672	1 mM	K21	A16	SBC4, SBC8
945.675	3779.676	1 mM	K148	S23, S24	BS^2^G
975.254	4872.240 (3 Met oxid.)	30 nM/1 mM	K75	L1	SBC4, SBC8

### 3D-Structure of the CaM/skMLCK Complex

Identification of cross-linked products between CaM and the skMLCK peptide was performed by nano-HPLC/nano-ESI-LTQ-Orbitrap-MS/MS (Figure 3). The products resulting from the cross-linking reaction with BS^2^G conducted at 1 mM Ca^2+^ are in agreement with the published NMR structure of the CaM/skMLCK peptide (M13) complex [13] as illustrated in Figures S4A, B in File S1 (see also Table S1 in File S1). Following earlier work, Cα–Cα distances up to 19 Å (or even 25 Å) can be assumed as constraints from chemical cross-linking experiments with reagents possessing spacer lengths of up to 11 Å, such as BS^2^G and SBC [18], [30]. Some slightly longer distances can be explained by CaM's inherent flexibility, especially in the α-helical linker region that connects both lobes. The amino acids of CaM that were found to be connected with the skMLCK peptide are comparable to those detected in a previous cross-linking study, in which Lys-21 and 30 in the *N*-terminal lobe of CaM as well as Lys-75 and 77 in the central α-helix, and Lys-94 in the *C*-terminal lobe of CaM had been cross-linked with the skMLCK peptide M13 [31]. Interestingly, several cross-linked products include the *C*-terminal amino acid of CaM (Lys-148), which is quite unusual compared to our previous experiences in studying CaM/peptide complexes [21], [31]. Figure S4C in File S1 shows the distances of cross-linked amino acids in the NMR structure of the CaM/Munc13-1 peptide complex [10], clearly indicating that this structure does not exist for the CaM/skMLCK peptide complex (PDB entry 2BBM). We analyzed intermolecular (between CaM and the skMLCK peptide) as well as intramolecular (within CaM) cross-linked products; yet, almost none of the cross-links were in agreement with the structure of the CaM/Munc13-1 peptide complex. This indicates that the complex between CaM and the *C*-terminally elongated skMLCK peptide is structurally different from the CaM/Munc13 peptide complex. Also at 30 nM Ca^2+^, the cross-links are consistent with the NMR structure of the CaM/M13 skMLCK peptide (Table S1 in File S1).

### 3D-Structure of the CaM/skMLCK F19A Complex

For the cross-linking reaction between CaM and skMLCK F19A peptide (Table S2 in File S1), a lower number of cross-links was found compared to the CaM complex with the skMLCK peptide (Table S1 in File S1). However, the detected cross-linked products lead to the same conclusion as for the skMLCK peptide (Figure S5 in File S1): Especially the cross-link between the *N*-terminal lobe of CaM (Lys-21) and skMLCK F19A peptide exhibits a distance in the structure of the CaM/Munc13-1 complex the cross-linker BS^2^G is not able to bridge. This clearly demonstrates that the unusual structure of the CaM/Munc13 peptide complex does not exist for the complex between CaM and skMLCK F19A peptide (Figure S5C in File S1). Apparently, removing the bulky, hydrophobic anchor amino acid at position 14 of the CaM binding motif, mimicking a 1-5-8-26 motif, does not induce a structure comparable to that of the CaM/Munc13 peptide complex.

### 3D-Structure of the CaM/skMLCK F19E Complex

Nano-HPLC/nano-ESI-LTQ-Orbitrap-MS/MS analysis led to the identification of a number of cross-linked products between CaM and the skMLCK F19E peptide (Table S3 in File S1). Almost all distances between cross-linked amino acids in CaM and skMLCK F19E peptide at 1 mM Ca^2+^ are in agreement with the structure of the CaM/M13 skMLCK peptide complex (Figures S6A, B in File S1). The few distances that are slightly longer than the expected maximum Cα-Cα distance of ca. 25 Å the cross-linkers are able to bridge can be explained by the inherent flexibility of CaM's central α-helix (Lys-75 and 77). Although the hydrophobic anchor amino acid Phe at position 14 of skMLCK's CaM binding motif is exchanged to an acidic glutamate, which present in the CaM binding motif of Munc13-1 (Figure 1), the unusual structure of the CaM/Munc13-1 peptide complex is apparently not induced by this amino acid exchange.

### 3D-Structure of the CaM/skMLCK F19E/L31W Complex

**Figure 5:**
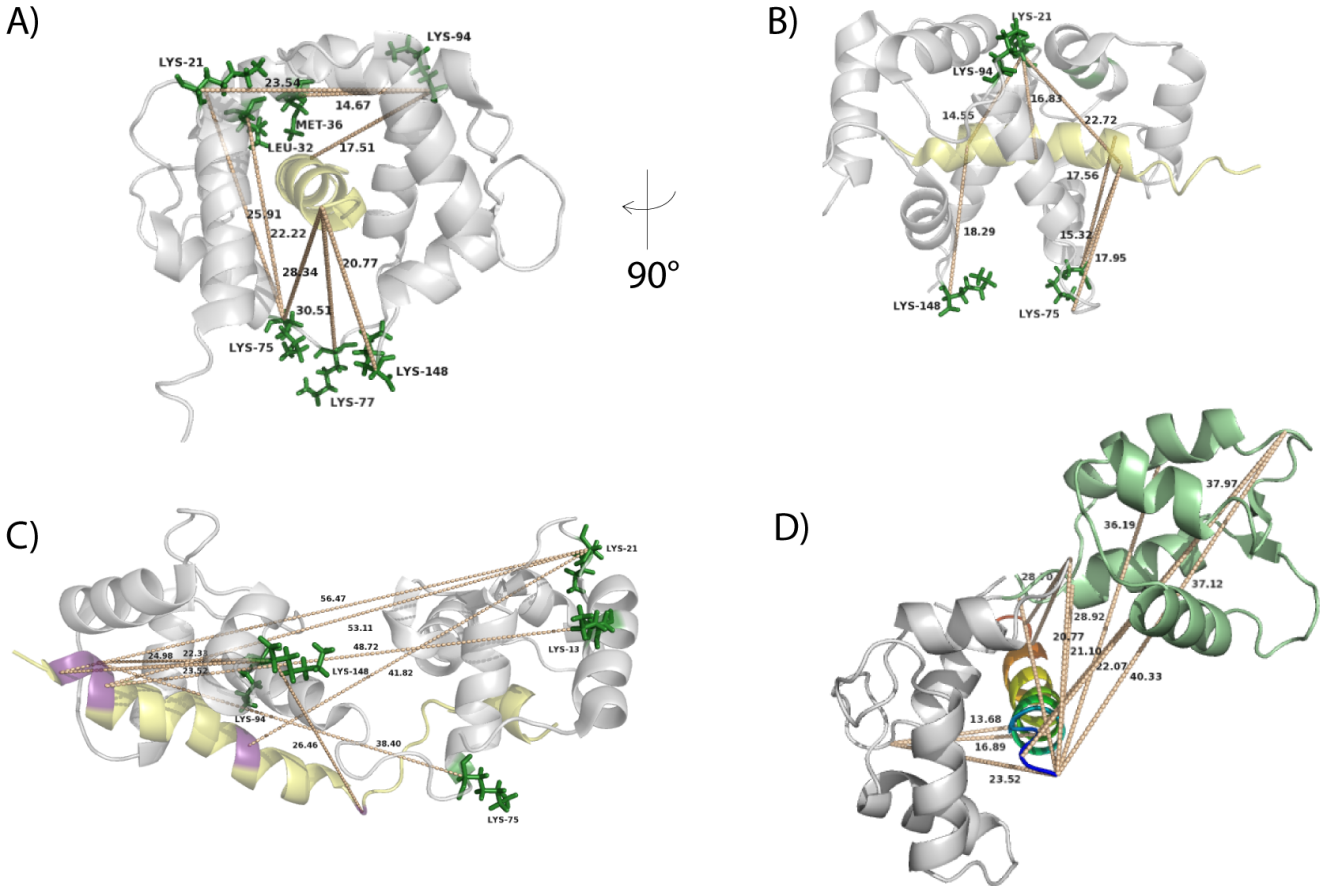
Identified cross-linked products between CaM and the skMLCK F19E/L31W peptide. Distances between cross-linked amino acids (at 1 ***mM*** Ca^2+^) are presented in the NMR structures of (A, B) CaM/M13 skMLCK peptide complex (PDB entry 2BBM, [13]; viewed from two angles and (C) CaM/Munc13-1 peptide complex (PDB entry 2KDU, [10]). CaM is colored in grey, the peptide is shown in yellow. Reacted amino acids in CaM are displayed as green sticks and those of the peptide are shown in purple. (D) Distances of cross-linked amino acids identified at 30 nM Ca^2+^ displayed in the half-loaded CaM (*N*-terminal domain of apo-CaM (PDB entry 2L53 [23], aa 1–77; green) and *C*-terminal domain of holo-CaM (PDB entry 2BBM [13], aa 78–147; grey)). The skMLCK F19E/L31W peptide is shown in rainbow colors. Distances (in Å) between Cα atoms of connected amino acids are illustrated as dotted lines.

To exclude that the extended conformation of CaM is exclusively formed in the presence of a bulky hydrophobic amino acid at position 26 of the CaM binding motif, we exchanged the leucine at position 31 of the peptide to tryptophan in order to mimic Munc13-1 (Figure 1). Analysis of the cross-links between CaM and the skMLCK F19E/L31W peptide resulted in the identification of a number of cross-linked products (Table 1 and Table S4 in File S1). As an example, the SBC-cross-linked product between Lys-94 of CaM and Leu-1 of skMLCK F19E/L31W peptide is presented in Figure 4. Both lysine residues in the *C*-terminal (Lys-94, Lys-148) and *N*-terminal CaM domains (Lys-13, Lys-21) had reacted with the *N*-terminus of the skMLCK F19E/L31W peptide (Leu-1, Met-2, Lys-3, Arg-4) indicating a collapsed conformation of CaM in the complex (Figure 5). Apparently, not even a skMLCK peptide that highly resembles the amino acid sequence of the CaM binding motif of Munc13 can induce an extended structure comparable to that of the CaM/Munc13 peptide complex (PDB entry 2KDU).

### Surface Plasmon Resonance (SPR) Spectroscopy

**Figure 6:**
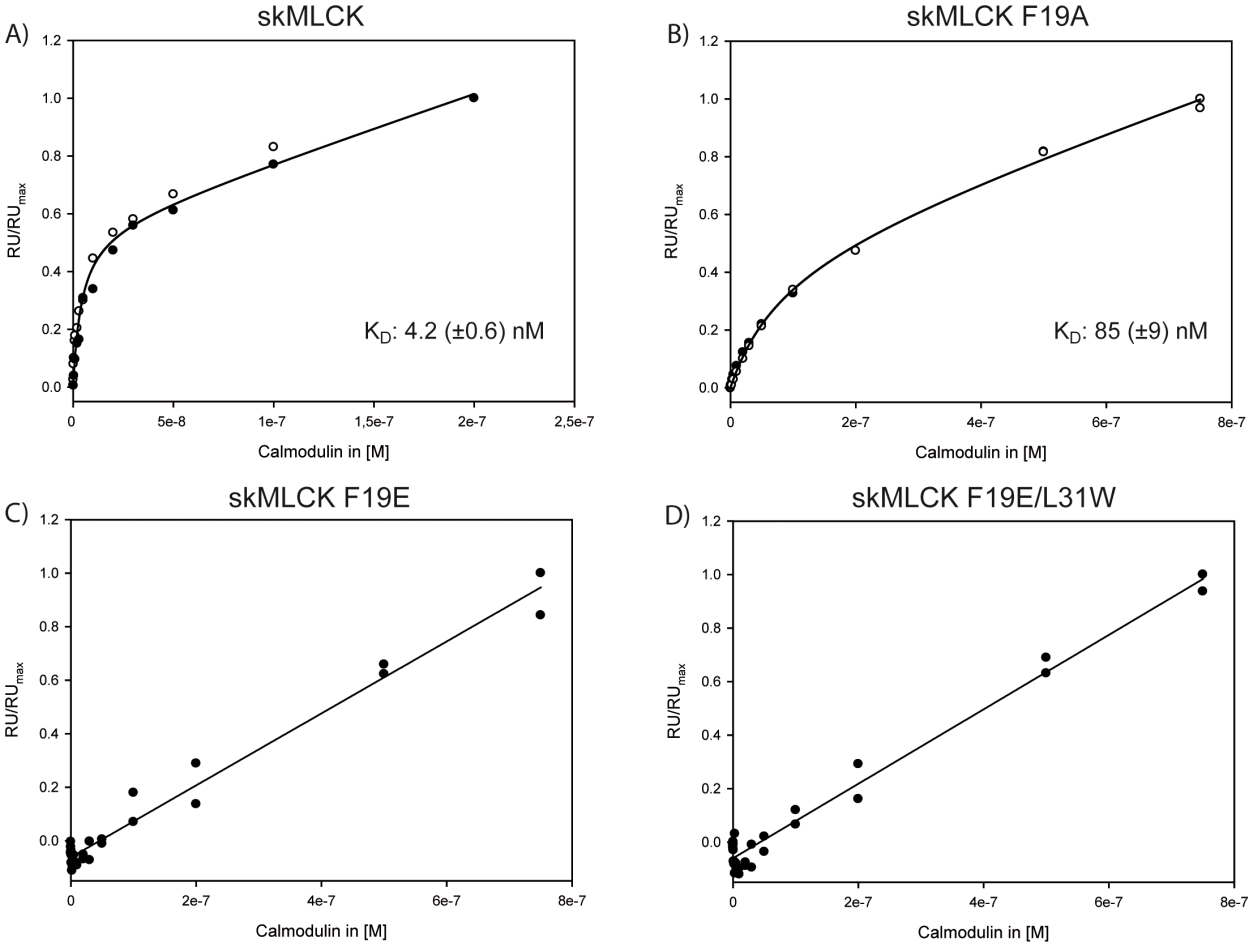
SPR analysis of CaM binding to skMLCK peptides. The CaM-dependent steady-state response is shown for (A) skMLCK peptide, (B) skMLCK F19A, (C) skMLCK F19E, and (D) skMLCK F19E/L31W. Solid lines indicate the results of fitting the data according to eq (1) (for (A) and (B)) or a linear fit (for (C) and (D)). Dissociation constants (K_D_) with standard errors are given for (A) and (B) resulting from two independent measurements (open and filled circles). RU: response unit; RU_max_: maximum response unit reached during the measurement.

In addition to the cross-linking experiments, we performed SPR analysis to characterize the binding of skMLCK peptide variants to CaM. The dissociation constant (K_D_) of the skMLCK M13 peptide for CaM binding is in the low nanomolar range [32], which is comparable to that of the Munc13-1 peptide as proposed by NMR titration experiments [10]. The *C*-terminally elongated skMLCK peptide used in our study also exhibits a K_D_ value in the low nanomolar range (4.2 (±0.6) nM, Figure 6A), showing that the additional *C*-terminal residues of the skMLCK peptide do not influence its CaM binding behavior. Besides this high-affinity phase, the CaM-dependent steady-state response revealed a second linear phase, indicating a low-affinity binding event. In previous studies, low-affinity binding of a second peptide to CaM had been observed for Munc13 peptides [7], [8], suggesting the presence of an alternative target binding region in CaM. In contrast, the phenylalanine residue at position 14 of the CaM binding motif is crucial for skMLCK binding, as evidenced by the K_D_ of skMLCK F19A (85 (±9) nM, Figure 6B). Apparently, the exchange of the hydrophobic anchor amino acid phenylalanine to an acidic glutamate - resembling the CaM binding motif of Munc13-1 - dramatically reduces the CaM affinity of this skMLCK variant. Here, only the low-affinity binding event is observed, while the high-affinity binding event is no longer detectable in the concentration range investigated herein (Figure 6C). A similar binding behavior is observed for the skMLCK variant with an additional exchange (Leu-31→Trp) (Figure 6D), suggesting that the tryptophan introduced at position 26 of the CaM binding motif cannot act as a second anchor residue.

## Discussion

We investigated the 3D-structures of complexes between CaM and skMLCK peptide variants mimicking the CaM binding motif of Munc13 by chemical cross-linking in combination with mass spectrometry. In order to mimic the unique 1-5-8-26 CaM motif of Munc13-1, we exchanged the hydrophobic anchor amino acid Phe-14 of the CaM-binding motif in skMLCK to alanine and glutamate, respectively. Additionally, the leucine residue at position 26 of the motif was changed to tryptophan, creating a 1-5-8-26 motif similar to that of Munc13-1. We show that the distance constraints imposed by the chemical cross-links in all CaM/skMLCK peptide complexes agree well with the published NMR structure of the CaM/M13 skMLCK peptide complex (PDB entry 2BBM) [13]. In this structure, CaM is collapsed around the peptide, which brings the two lobes close to each other (Figures 5A, B, Figures S4A, B; S5A, B; S6A, B in File S1) and allows bridging the distances between *N*- and *C*-terminal CaM domains by both cross-linkers used herein. Compared to this collapsed form, the conformation of CaM in the CaM/Munc13-1 peptide complex (PDB entry 1KDU) is more extended [10]. Here, the distances between *N*- and *C*-terminal lobes of CaM are much longer, making it impossible for both cross-linkers to connect amino acids between the two CaM domains. The cross-links found at a high Ca^2+^ concentration consistently point to the CaM/M13 skMLCK peptide structure (Figures 5A and B; Figures S4A, B; S5A, B; S6A, B in File S1). At Ca^2+^ concentrations in the nanomolar range, CaM is only half-saturated with Ca^2+^. In order to investigate whether the cross-links found at 30 nM Ca^2+^ are in agreement with a half Ca^2+^-loaded CaM structure, we docked the *N*-terminus of apo-CaM (PDB entry 2L53, [23]) with the *C*-terminus of holo-CaM (PDB entry 2BBM, [13]). The majority of cross-links are consistent with this half Ca^2+^-loaded CaM structure indicating that CaM might adopt a similar conformation, i.e., a mixed apo-/holo-form, when binding to the skMLCK peptide at low Ca^2+^concentrations (Figure 5D). On the other hand, our cross-linking data do not exclude the possibility that the CaM/skMLCK (M13) peptide complex exhibits identical structures at low and high Ca^2+^ concentrations. Overall, our cross-linking results do not support the extended conformation of CaM that is present in the complex with Munc13-1 - even if the CaM binding motif of skMLCK is changed to highly resemble that of Munc13-1 [10].

Furthermore, we performed SPR measurements to determine the CaM binding affinities of the skMLCK peptide variants used herein. We found a reduced CaM affinity for the skMLCK F19A peptide (Figure 6B) where the bulky phenylalanine is exchanged to alanine. The CaM binding affinity is drastically reduced if this phenylalanine is exchanged to glutamate (Figure 6C) and if the leucine at position 31 is additionally exchanged to tryptophan (Figure 6D). Recently, a general model was proposed for the conformation CaM can adopt in its complexes, relying on the distance between the first and second bulky hydrophobic anchor residue [33]. This model implies that a continuous α-helix is created between the two anchor amino acids. In case of Munc13 peptides, a hairpin structure is formed, underlining the atypical character of the 1-5-8-26 CaM binding motif present in Munc13. Intriguingly, we did not identify a 1-5-8-26 motif comparable to that of Munc13-1 when mining a CaM target database (*32*) and a recent protein array screen of neuronal CaM-binding proteins (*33*). Therefore, we conclude that the 1-5-8-26 CaM binding motif of Munc13 is unique and induces an unusual conformation of the complex, i.e., an extended structure with a modular architecture. At least for Munc13-1 and ubMunc13-2, this is likely to be the structural basis for a sequential CaM binding mode (*10*), and as such for the ability of Munc13-CaM complexes to sense Ca^2+^ over a broad concentration range.

## Supporting Information

File S1 **Figure S1, CD experiments with different TFE concentrations.** (0–50%) of A) skMLCK peptide, B) skMLCK F19A, C) skMLCK F19E, D) skMLCK F19E/L31W. **Figure S2, SDS-PAGE of cross-linking experiments of CaM and skMLCK, skMLCK F19A, and skMLCK F19E.** Cross-linking reactions were carried out at a 50-fold molar excess of BS2G with a Ca^2+^ concentrations 30 nM. Lane 10: As a control, CaM was diluted in water without adding buffer, EDTA, or Ca^2+^; please note the different Ca^2+^-loaded states of CaM. M: protein marker. **Figure S3, MALDI-TOF mass spectrum (MS) of the cross-linking reaction mixture between CaM and skMLCK F19E peptide (30 min incubation time, 1 mM Ca^2+^, 50-fold molar excess of BS^2^G).** Singly (*m/z* range 16,000–21,000) and doubly (*m/z* range 8,000–11,000) charged ions of non-cross-linked CaM modified with partially hydrolyzed cross-linker molecules and CaM/skMLCK (1∶1) peptide complexes are visible. XL: cross-linker; n.a.: not assigned. **Figure S4, Identified cross-linked products (cross-linkers BS^2^G and SBC) between CaM and the skMLCK peptide at 1 mM Ca^2+^.** Distances between cross-linked amino acids are presented in the NMR structures of (A, B) the CaM/M13 kMLCK peptide complex (PDB entry 2BBM, [13]; viewed from two angles) and (C) the CaM/Munc13-1 peptide complex (PDB entry 2KDU, [10]). CaM is colored in grey, the peptide is shown in yellow. Reacted amino acids in CaM are displayed as green sticks, reacted amino acids in the peptide are shown in purple. Distances (in Å) between Cα atoms of connected amino acids are shown as dotted lines. **Figure S5, Identified cross-linked products (cross-linkers BS^2^G and SBC) between CaM and the skMLCK F19A peptide at 1 mM Ca^2+^.** Distances between cross-linked amino acids are presented in the NMR structures of (A, B) the CaM/M13 skMLCK peptide complex (PDB entry 2BBM, [13]; viewed from two angles) and (C) the CaM/Munc13-1 peptide complex (PDB entry 2KDU, [10]). CaM is colored in grey, the peptide is shown in yellow. Reacted amino acids in CaM are displayed as green sticks, reacted amino acids in the peptide are shown in purple. Distances (in Å) between Cα atoms of connected amino acids are shown as dotted lines. **Figure S6, Identified cross-linked products (cross-linkers BS^2^G and SBC) between CaM and the skMLCK F19E peptide at 1 mM Ca^2+^.** Distances between cross-linked amino acids are presented in the NMR structures of (A, B) the CaM/M13 skMLCK peptide complex (PDB entry 2BBM, [13]; viewed from two angles) and (C) the CaM/Munc13-1 peptide complex (PDB entry 2KDU, [10]). CaM is colored in grey, the peptide is shown in yellow. Reacted amino acids in CaM are displayed as green sticks, reacted amino acids in the peptide are shown in purple. Distances (in Å) between Cα atoms of connected amino acids are shown as dotted lines. **Scheme S1, Structures and spacer lengths of cross-linkers used in this study. Table S1, Summary of cross-linked products (intra- and intermolecular) for CaM and skMLCK peptide.** For SBC, irradiation energies are indicated (4 or 8 J/cm^2^. **Table S2, Summary of cross-linked products (intra- and intermolecular) for CaM and skMLCK F19A peptide.** For SBC, irradiation energies are indicated (4 or 8 J/cm^2^). **Table S3, Summary of cross-linked products (intra- and intermolecular) for CaM and skMLCK F19E peptide.** For SBC, irradiation energies are indicated (4 or 8 J/cm^2^). **Table S4, Summary of cross-linked products (intra- and intermolecular) for CaM and skMLCK F19E/L31W peptide.** For SBC, irradiation energies are indicated (4 or 8 J/cm^2^).
(DOCX)
