# Supplementary material for: Munc13-Like skMLCK Variants Cannot Mimic the Unique Calmodulin Binding Mode of Munc13 as Evidenced by Chemical Cross-Linking and Mass Spectrometry
Source: PLoS One. 2013 Oct 10;8(10):e75119. doi: 10.1371/journal.pone.0075119 (PMC3794949; doi:10.1371/journal.pone.0075119)
Supplement: File S1 — Figure S1, CD experiments with different TFE concentrations. (0–50%) of A) skMLCK peptide, B) skMLCK F19A, C) skMLCK F19E, D) skMLCK F19E/L31W. Figure S2, SDS-PAGE of cross-linking experiments of CaM and skMLCK, skMLCK F19A, and skMLCK F19E. Cross-linking reactions were carried out at a 50-fold molar excess of BS2G with a Ca2+ concentrations 30 nM. Lane 10: As a control, CaM was diluted in water without adding buffer, EDTA, or Ca2+; please note the different Ca2+-loaded states of CaM. M: protein marker. Figure S3, MALDI-TOF mass spectrum (MS) of the cross-linking reaction mixture between CaM and skMLCK F19E peptide (30 min incubation time, 1 mM Ca2+, 50-fold molar excess of BS2G). Singly (m/z range 16,000–21,000) and doubly (m/z range 8,000–11,000) charged ions of non-cross-linked CaM modified with partially hydrolyzed cross-linker molecules and CaM/skMLCK (1∶1) peptide complexes are visible. XL: cross-linker; n.a.: not assigned. Figure S4, Identified cross-linked products (cross-linkers BS2G and SBC) between CaM and the skMLCK peptide at 1 mM Ca2+. Distances between cross-linked amino acids are presented in the NMR structures of (A, B) the CaM/M13 kMLCK peptide complex (PDB entry 2BBM, [13]; viewed from two angles) and (C) the CaM/Munc13-1 peptide complex (PDB entry 2KDU, [10]). CaM is colored in grey, the peptide is shown in yellow. Reacted amino acids in CaM are displayed as green sticks, reacted amino acids in the peptide are shown in purple. Distances (in Å) between Cα atoms of connected amino acids are shown as dotted lines. Figure S5, Identified cross-linked products (cross-linkers BS2G and SBC) between CaM and the skMLCK F19A peptide at 1 mM Ca2+. Distances between cross-linked amino acids are presented in the NMR structures of (A, B) the CaM/M13 skMLCK peptide complex (PDB entry 2BBM, [13]; viewed from two angles) and (C) the CaM/Munc13-1 peptide complex (PDB entry 2KDU, [10]). CaM is colored in grey, the peptide is shown in yellow. Reacted amino acids in C [file pone.0075119.s001.docx]

**Supplemental Material**

Scheme S1

| *Name* | *Chemical Structure* | *Spacer Length* |
| --- | --- | --- |
| BS^2^G-*D_0_*/*D_4_* | *Bis*(sulfosuccinimidyl)glutarate   | 7.7 Å |
| SBC | *N*-succinimidyl-*p*-dihydrobenzoylcinnamate   | 10.2 Å |

Figure S1


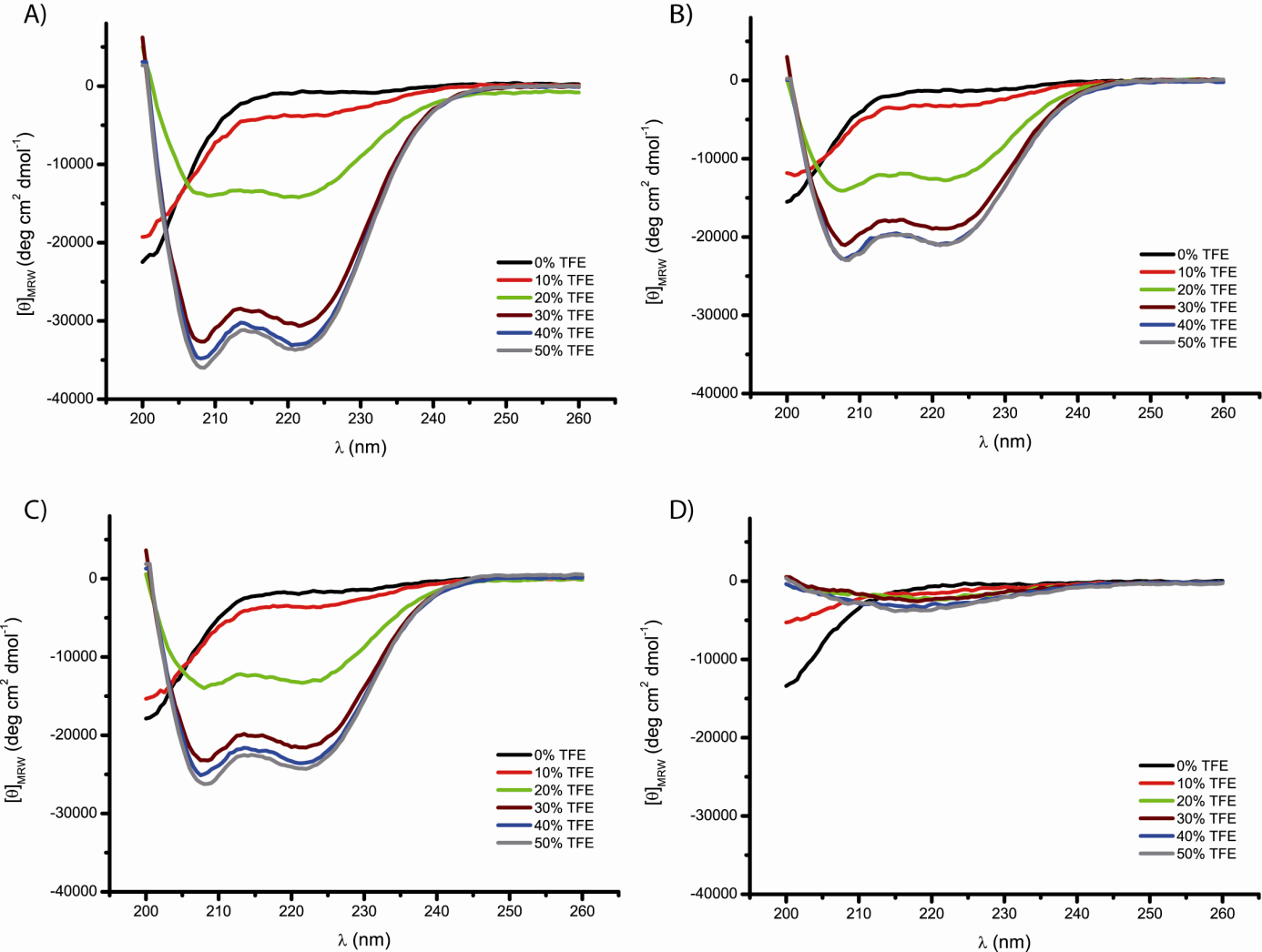


Figure S2


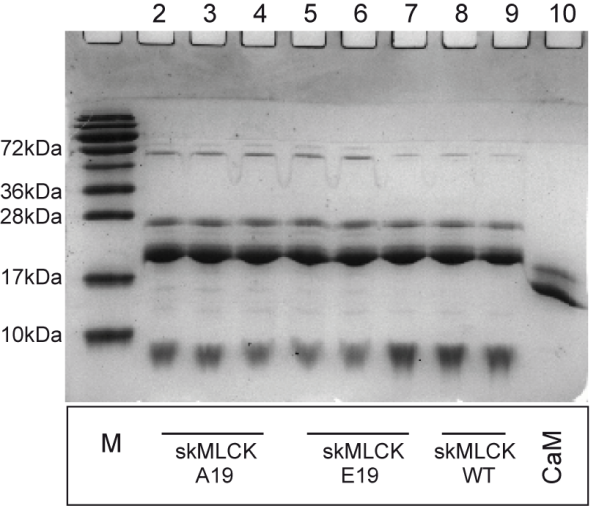


Figure S3

Figure S4

Figure S5

Figure S6

Table S1

Table S2

Table S3

Table S4
